# IoT-Based Microclimate and Vibration Monitoring of a Painted Canvas on a Wooden Support in the Monastero of Santa Caterina (Palermo, Italy)

**DOI:** 10.3390/s22145097

**Published:** 2022-07-07

**Authors:** Carlo Trigona, Eliana Costa, Giuseppe Politi, Anna M. Gueli

**Affiliations:** 1 Dipartimento di Ingegneria Elettrica Elettronica e Informatica (DIEEI), University of Catania, Viale Andrea Doria 6, 95125 Catania, Italy; eliancost@gmail.it; 2 Dipartimento di Fisica e Astronomia “Ettore Majorana” (DFA), University of Catania, Via Santa Sofia 64, 95123 Catania, Italy; giuseppe.politi@unict.it (G.P.); anna.gueli@unict.it (A.M.G.)

**Keywords:** IoT measurement system, microclimate monitoring, vibrations, structural health monitoring, preventive conservation, cultural heritage preservation

## Abstract

The main objective of this work is the characterization and observation of the performance of an IoT measurement and monitoring system in the field of cultural heritage conservation for assessing the health condition of artworks. This article also describes the application of this system to the monitoring of a canvas painting applied on a wooden support, an artwork from the 19th century by the painter Giuseppe Patricolo depicting *The Deposition*, placed inside a niche in the Santa Caterina Monastery in Palermo (Italy). Considering the presence of the wooden structure, it is useful to measure not only microclimatic parameters such as temperature and humidity, but also vibrations that can in fact cause degradation phenomena in these artworks. This is a first step towards the development of mimetic systems integrated in the work of art without causing physical, mechanical or chemical alterations and ensuring that the level of microclimatic parameters is below the threshold values whose exceeding could compromise the entire artefact.

## 1. Introduction

Most of the interventions of restoration and conservation of works of art implies very often a number of operations that can be complex, expensive although the special talent of the restorer can be considered an unmeasurable value, and it could be dangerous. A wrong intervention could in fact compromise an artwork irreversibly [1,2,3,4]. The ever-increasing technical and scientific accuracy nevertheless involves a strong economic commitment, even more so when, according to a practice that is not always feasible, a punctual diagnostic campaign is carried out [5,6]. The intervention of restoration is inevitable and, in this context, the persistence of factors that initiate and/or accelerate the phenomena of degradation on cultural heritage inevitably leads to the decay of the material. At that point, after the restoration operations have had their successful outcome, it would be desirable to implement and maintain ordinary scheduled maintenance interventions, which would therefore prolong the beneficial effects [7,8]. From this point of view, a far-sighted and conscious attitude is spreading, especially in Anglo-Saxon and Northern European countries, where it is well-consolidated that preventive conservation represents a series of acts and programs whose aim is to maintain the physical characteristics and some microclimate factors within tolerable values, preventing or controlling the development of potentially harmful conditions [9,10,11]. The physical quantities of interest that most influence a good conservation of artistic artifacts are relative humidity, temperature, vibrations, illuminance and pollution [12,13,14,15,16]. In particular, the thermo-hygrometric variations, often together with vibrations, produce on every material more or less evident phenomena of expansion or contraction, which constitute an important stress; this is particularly true for hygroscopic materials such as wood and canvas of natural vegetable fiber [17,18,19,20,21,22,23]. A mixed system as canvas on a wooden frame is even more delicate and susceptible to different behaviors with changes in humidity, temperature and kinetic sources such as induced vibrations; in fact, the wood expands anisotropically, whereas the canvas, at least up to a certain RH, shrinks because the fibers and yarns swell transversally with an increase in tension. For this reason, as it often occurs, the slippage of the fibers within the yarn produces a macroscopic deformation of the fabric that manifests itself with a lower tension or a total relaxation. If the tension exceeds the elastic limit, the deformation becomes plastic (permanent). Humidity can interact with the artifact by changing its size, as well as the speed and type of chemical reactions that a material lives; moreover, it constitutes an ideal environment for the proliferation of biological and microbiological degradation phenomena [23,24,25]. Natural organic materials such as wood, textiles (e.g., the textile support of paintings) and glues used for the preparatory layers, plaster or pigments can react with swelling and expansion. Humidity can also accelerate or initiate metal corrosion and is generally a crucial factor in the preservation of textiles. Temperature can cause several degradation phenomena, especially when there is an increase in this factor; the first effect is an acceleration of chemical reactions, proportional to the increase in temperature. Moreover, the oscillations of this parameter are reflected in the humidity trend, causing movements such as expansion or shrinkage and embrittlement. For this reason, it is of extreme interest to measure physical quantities such as relative humidity, temperature and vibrations, considering that all of them could contribute to the degradation of artifacts [26]. In order to perform measurements of these physical quantities, various systems and sensors have been proposed in literature and each solution is strictly correlated with the specific application and artifact monitoring. In fact, the rapid technological advancement of IoT-based measurement systems for temperature, humidity and vibrations has encouraged researchers to create smart solutions and novel sensing elements able to record specific signal in sites of historical and artistic interest. This activity includes micro-climate monitoring systems for cultural heritage structures and monuments [27,28,29]. In [30], the authors present a wireless sensor network for the distributed monitoring for artifacts. The proposed custom solution is based on an IoT measurement node (2.5 cm × 1.5 cm), with wireless communication connected to the Internet with data storage for remote management. The system is able to measure temperature and humidity with an architecture based on a nRF51822 SoC supplied through a battery of 3.3 V. In [31], the authors propose an environmental monitoring device composed on an array of piezoelectric quartz crystals as mass sensor, temperature and relative humidity sensor. It is a custom solution based on a sensirion sensor (SHT75), with a data transmission through standard IEEE 802.15.4 and a memory card. The system is supplied through a lithium battery and in order to decrease the power supply a wake-up solution is considered. The system was applied at the Apsley House in London and at the Royal Palaces of Abomey in Benin. In [32], the authors propose a low-cost IoT-based wireless sensor network for the environmental monitoring of cultural heritage. The solution regards a MEMS SiP, BNO055, as a PCB prototype able to measure tilt and shock. A 5 V source is used to supply the device which includes a radio module ZigBee unit IEEE 802.15.4 in 2.4 GHz band, which is suitable for various museum monitoring. In [33], an open-source hardware for measurement of microclimate parameters is presented. In particular, in this work, the relative humidity and temperature are monitored inside the Mosque-Cathedral of Córdoba through the adoption of microclimate stations mainly located inside of the heritage. Each measurement system is based on an Arduino single-board microcontroller, in order to estimate both the quantities of interest. The commercial sensor is represented by DHT22 with a supply voltage with a battery of 5 V and a I2C communication protocol. It is worth noting the interest of IoT is clearly felt in the field of cultural heritage [34] for the ability to access information from anywhere at any time on any device, to improve communication between connected electronic devices, to transfer data over a connected network and to reduce human intervention during the estimation of the microclimate parameters.

In this paper, in order to monitor temperature, humidity and vibration in a canvas painting on a wooden support, placed in the Monastero of Santa Caterina (Palermo, Italy), an IoT-based measurement system based on a STEVAL-MKSBOX1V1 board was tested. The novelty of the activity is correlated with the artwork considered and with its monitoring that, for the first time, has been implemented with a smart measurement system.

The system is capable of communicating with the ST BLE Sensor app and a smartphone connects via Bluetooth. The entire system is supplied through a 3.3 V battery. It is able to perform the measurement of the three microclimate parameters by using STTS751 of interest with a data logger. A Digital temperature sensor (STTS751), a 3-axis accelerometer (LIS2DW12) and a humidity sensor (HTS221) were used during the characterization and the test in the monastery. The main objective of this work is the characterization and observation of the performance of this device as a monitoring system for assessing the health condition of artworks. The proposed solution improves the state of the art considering the compactness of the system and at the same time the ability to measure multiple physical quantities managed through a low power IoT approach. It is worth nothing that no other work presents all these prerogatives.

## 2. Method

The management of museum sites, from usage to maintenance and planning of conservation and restoration, involves a series of operations that affect the site, the exhibition rooms and the individual artworks. Starting from the material and formal condition of the place, the maintenance of the cultural heritage in the best possible conditions is the work of clever design and sometimes massive and expensive operations; the most recent and shared international trend sees preventive conservation as the best strategy for the prediction and prevention of events that may accelerate or cause various phenomena of degradation, whether physical, chemical, biological or anthropic.

Knowledge of the work of art is an essential element for planning the ideal conditions, since each material has its own conservation requirements, given the particular reactivity of the constituent materials in relation to environmental and micro-environmental factors. The first step is to analyze the materials and the techniques used, then to study the microclimate, lighting and air quality parameters of the environment in which the work is located.

The data collected allows a modulation of the preventive strategies in terms of temperature (°C), relative humidity RH (%), illuminance (lux) and air quality control, focusing on the level of aerodisperse chemical pollutants, and also in relation to pest management.

As already mentioned in the previous sections, the measurement of temperature and relative humidity is crucial as these two parameters strongly influence the physical balance with the artefact relates to the environment, which is decisive for its conservation. In addition to that, the control of the level of illumination, according to the typical photosensitivity of each class of artefact, the measurement of the level and quality of airborne chemical pollutants, and the monitoring of possible biological attacks prevents potential damage to the artefact.

The development of small-scale, nanomaterials and structures can be decisive in measuring degradation phenomena before they can prove to be irreversibly destructive; nano-sized sensors and non-invasive devices can in fact be placed close to or “inside” the work of art, in order to measure some essential parameters and assess some details related to the state of conservation (e.g., nanosensors for measuring pH, dinanometric nanosensors for measuring dimensional variations or internal movements, nanosensors for measuring the ionic charge and quality of salts, etc.). The design and realization of a digital platform for dialogue, in which the detected data can be collected and associated to limit alarm values and in which it is possible to read simultaneously the different environmental parameters, can show the interaction between the different measurements and help trace the cause of a harmful unbalance in the control of the whole system.

These limit values are specified in national and international legislation on preventive conservation, which is more of a guideline than an actual prescription. The Ente Italiano di Normazione (UNI), for example, a national organism considered to be of reference for the cultural heritage field at international level (many ISO standards are based on Italian standards), has published several technical standards. These standards have the merit of providing practical answers to problems linked to the measurement of environmental parameters in relation to the materials conserved, but also have the limitation of setting “recommended values” that are poorly adaptable to the specific nature of each work of art and the environment in which it is located [35,36,37,38,39,40,41].

For this reason, any study in the field of preventive conservation must start from the specificity of the environment and the artwork. In Great Britain, for example, the Museums and Galleries Commission has adopted “Accreditation Standards”, which establish actions to reduce the risk of damage to the works. As far as microclimate control is concerned, there is a need to implement environmental monitoring to alert staff about potentially dangerous environmental conditions through the measurement of relative humidity, temperature and illuminance levels using simple or sophisticated instrumentation. This goal can only be achieved by multidisciplinary teams carrying out scientific research, field projects, training and dissemination of information in the field of preventive conservation.

The conservation of cultural heritage is ensured through a coherent, coordinated and programmed activity of study, prevention, maintenance and restoration. By prevention, we refer to the set of activities aimed at limiting risk situations related to the context. Maintenance indicates all the activities and interventions aimed at controlling the condition of the cultural heritage and maintaining the integrity, functional efficiency and identity of the assets and their components.

**Figure 1:**
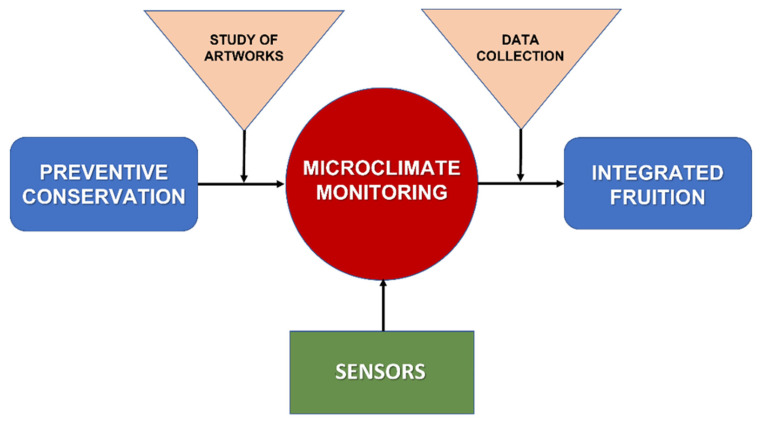
Description of the approach underlying the research: microclimate monitoring is the base of preventive conservation. In this context the study aimed at understanding the artwork has a fundamental role. The data collected by the sensors of the microclimatic parameters and the information obtained with the diagnostic techniques allow the integrated fruition.

The research presented in this work is based on an approach that considers microclimatic monitoring as playing a central role between the preventive conservation of a work of art and its integrated fruition (Figure 1). Any action aimed at preventing or reducing the degradation of a work of art can be defined as preventive conservation but cannot be separated from an extensive study of the artwork itself. This study must be based on historical sources, photographic documentation and diagnostic techniques for knowing the materials and the executive techniques. The continuous collection of data for microclimatic monitoring, performed with specific sensors for temperature, relative humidity, illuminance, UV radiation, vibrations and pollutants measurements, together with the collection at regular intervals of information obtained through diagnostics, such as chemical composition, material characterization, color specification, etc., makes integrated fruition possible. This latter is not only intended as observation and contemplation of artwork but also as the accessibility of historical-artistic information and diagnostic data useful to the public for the material knowledge and to specialists for the implementation of conservation and any restoration programs.

Microclimate monitoring was performed considering material-specific parameters relating to exposed artifacts. Example of reference values, extracted from UNI 10829:1999 [36] is reported in Table 1.

## 3. Experimental Results and Discussion

The STMicroelectronics STEVAL-MKSBOX1V1 was used as a wireless IoT-based device for measurement of the microclimate parameters. The data are transmitted through Bluetooth^®^ low energy. In particular, the temperature, humidity and vibrations were measured on a painting, realized by the painter Giuseppe Patricolo in the first half of 19th century and depicting *The Deposition*. Its structure is quite heterogeneous, with a painted canvas fixed to a wooden support by a thin layer of white mortar. The artwork, whose particular shape gives it a concave structure, is 3 m high and 2 m wide and is placed inside a niche in the Santa Caterina Monastery in Palermo (Italy).

**Figure 2:**
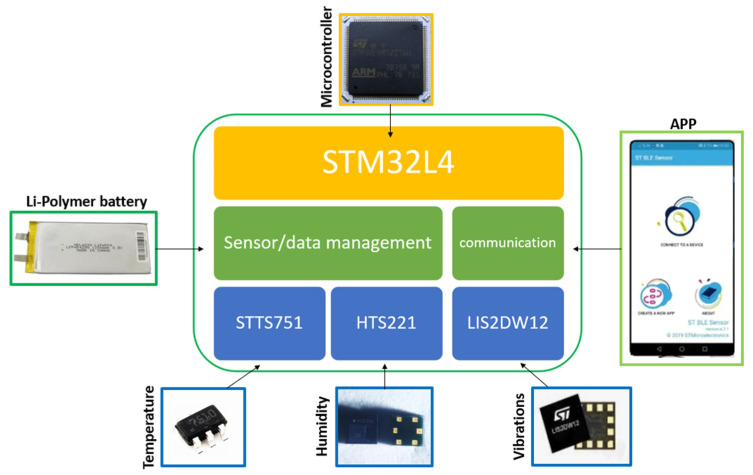
Schematic diagram of the IoT-based system for microclimate monitoring.

**Figure 3:**
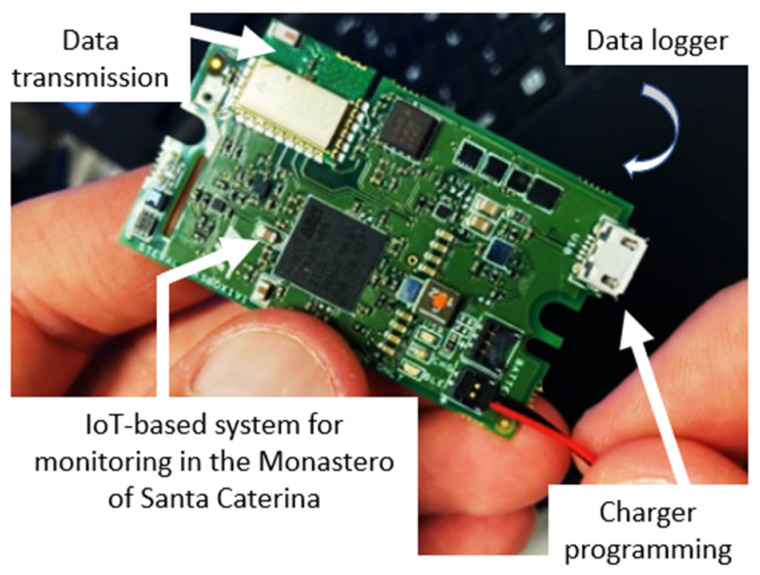
Board used for the monitoring of the painting.

The measurement system is schematized in Figure 2. As it can be noted, it presents various functionalities and the management of the measurands, the data logger and communication are accomplished through an ultra-low-power STM32L4R9 microcontroller. The system is able to manage the STTS751, used as temperature sensor, the HTS221, used as capacitive humidity sensor and the LIS2DW12, used as 3-axis accelerometers. Table 2 synthetizes the main features of the adopted sensors. A 3.3 V 1000 mAh Li-Polymer battery was used as a power supply of the measurement system. Figure 3 shows the board used for the monitoring of the painting. It should be noted that thanks to its compactness, performance in terms of data transmission, measurement approach and general features, this kind of sensor can be also applied to different exhibition spaces as well as directly applied on a large variety of specific works of art, e.g., painting on different supports, wooden frames statues and books.

**Figure 4:**
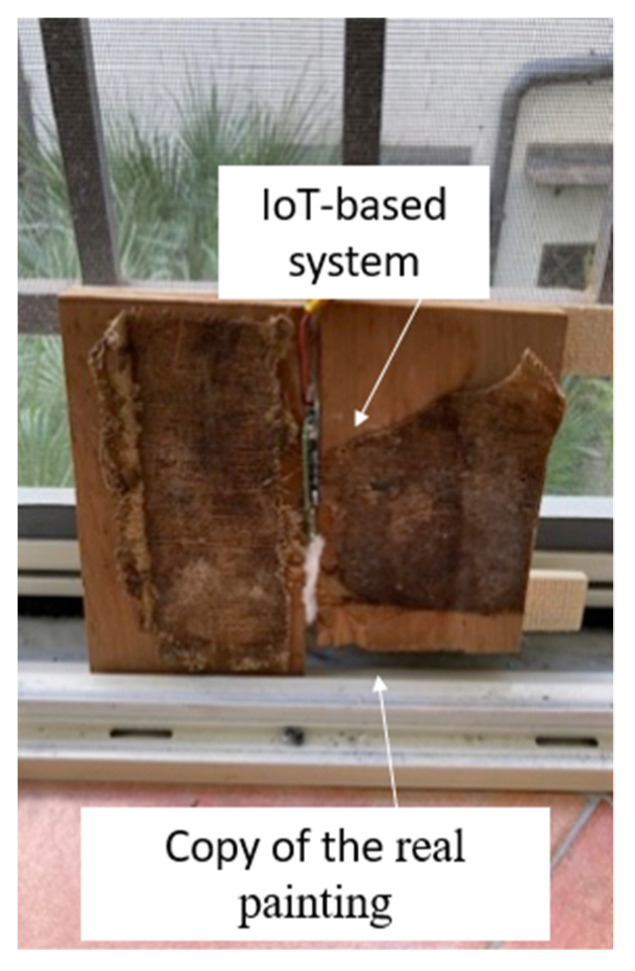
Prototype under test, copy of *The Deposition*.

In order to characterize the measurement system before testing the Giuseppe Patricolo’s painting for temperature, humidity and vibrations, a copy of a small part of the wooden support of the painting was realised (see Figure 4). This setup was used during the characterization in terms of vibrations, whereas, in order to characterize the board for measurements of temperature and humidity, the measurement system and its sensors were directly exposed to the controlled environment. This approach avoided unwanted contributions during characterization by the wood structure.

The device characterization was pursued by using a thermal chamber (ESPEC-SH-242) and an electrodynamic shaker (TIRA TV 50009) to impose known temperature, humidity and vibration levels, respectively. The chamber can operate in a temperature range from −40 °C to 150 °C and in a humidity range from 30% to 95% RH for temperatures higher than 15 °C. The main shaker specifications are: rated force 9 N, frequency range 2–20,000 Hz and max acceleration 60 g.

**Figure 5:**
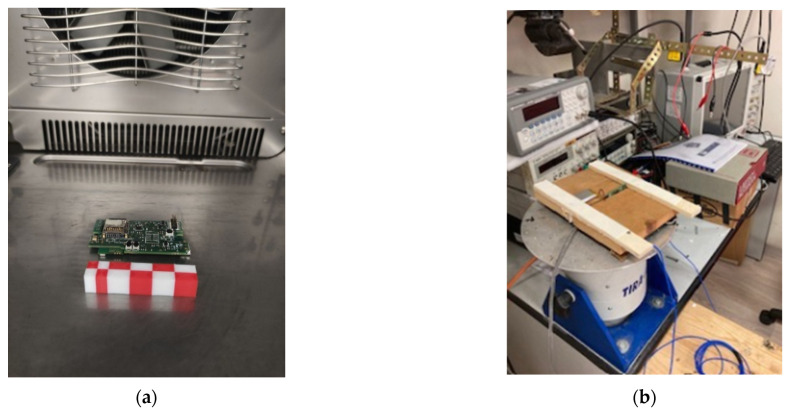
Experimental setup used in laboratory to characterize the IoT-based measurement system: (**a**) sensor inside climate chamber for relative humidity and temperature, (**b**) sensor on copy structure on the shaker.

Both the characterization setups are shown in Figure 5.

**Figure 6:**
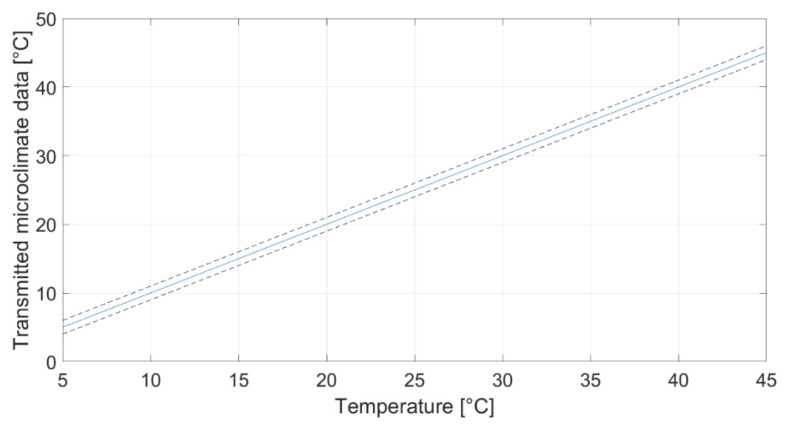
Characterization in terms of temperature at 50% of relative humidity.

**Figure 7:**
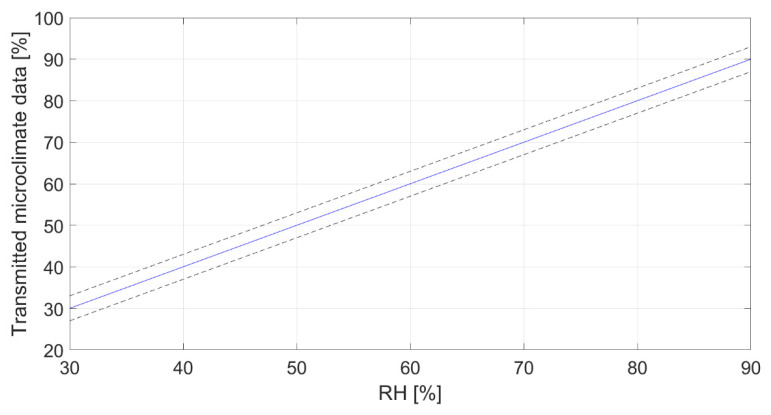
Characterization in terms of relative humidity at 20 °C.

**Figure 8:**
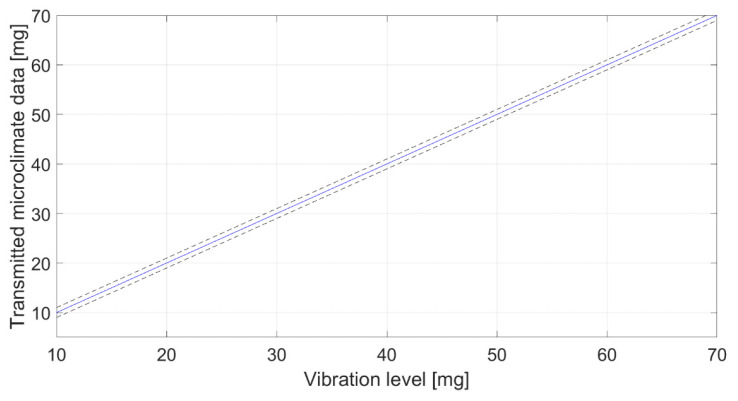
Characterization in terms of vibrations.

The characterization regarded the study of the data provided by the board as a function of the three parameters of interest set by the chamber and shaker. Results are presented in Figure 6, Figure 7 and Figure 8 for temperature (at 50% RH), relative humidity (at 20 °C) and acceleration, respectively. Each graph represents the calibration diagram able to correlate the transmitted output as a function of the measurand. Each one includes the mean value of the measurements (solid line) as a function of the imposed values, and the uncertainty (dotted lines), which appears in-line with the declared accuracy of the sensors (see Table 2).

Once the characterization of the device was completed, with the aim of testing it for microclimatic analysis, the measurements on the case study were accomplished inside the monastery.

**Figure 9:**
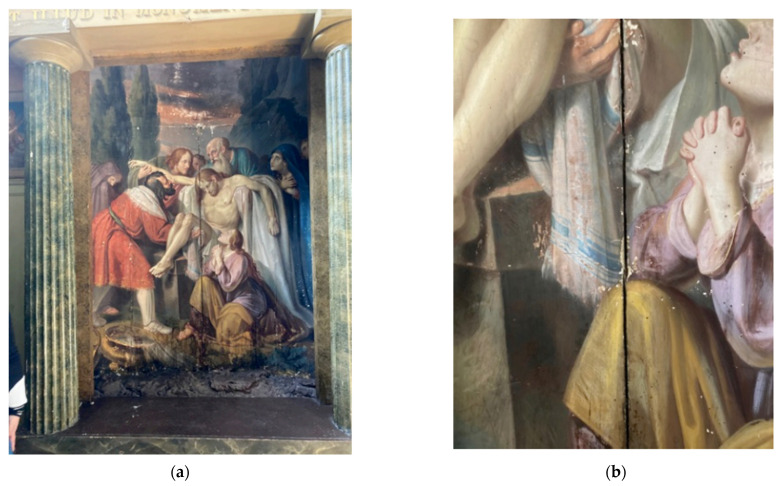
Microclimate monitoring system installed in the real painting (**a**) and a zoom (**b**) where the measurement system is installed inside the inlet.

The artwork and a zoom of the chosen area for the positioning of the device are presented in Figure 9. Measurement has been taken for a duration of about 12 h.

The system was placed in an inconspicuous area of the work to monitor the physical quantities of interest, and to compare the measured values with the threshold parameters described in the regulations [35,36,37,38,39,40,41].

The positioning in the open joint has been chosen to be hidden to the public as only one probe was available to collect microclimatic information about the entire space. It should be noted that vibration measurements could be useful in case of possible handling and transport.

**Figure 10:**
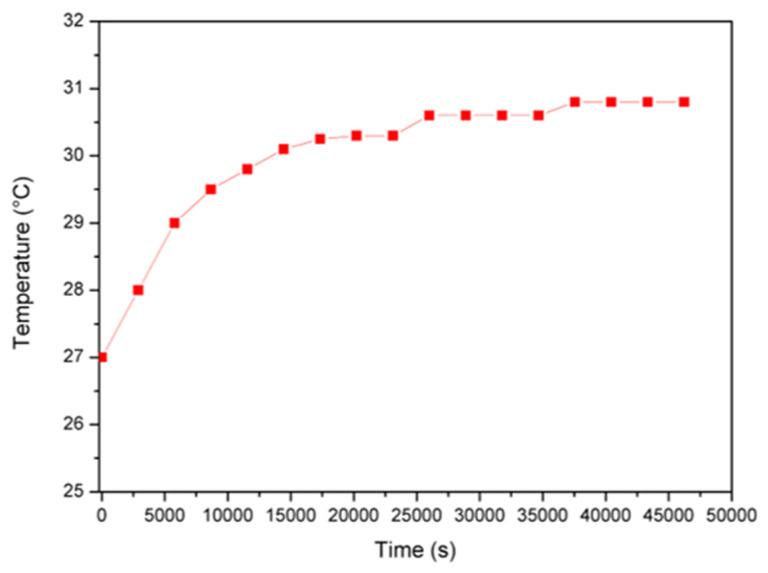
Evolution of the temperature obtained during the measurements campaign.

**Figure 11:**
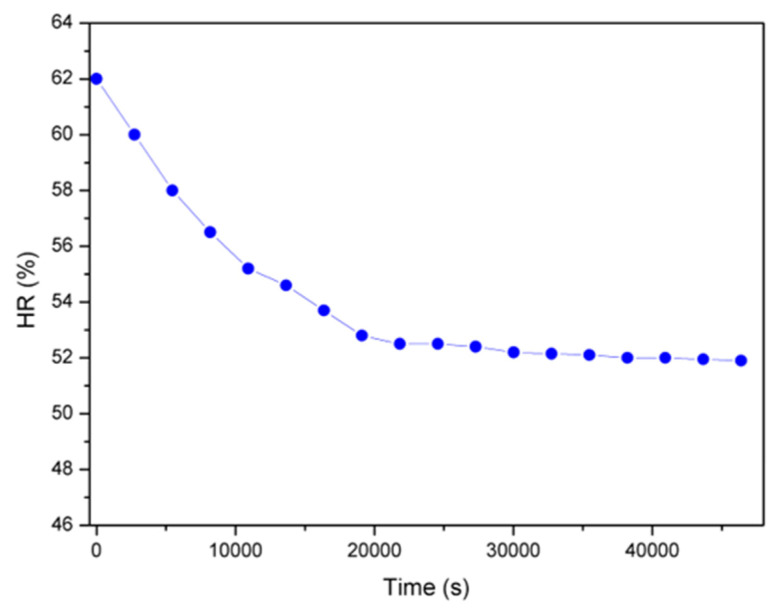
Evolution of the relative humidity obtained during the measurement campaign.

**Figure 12:**
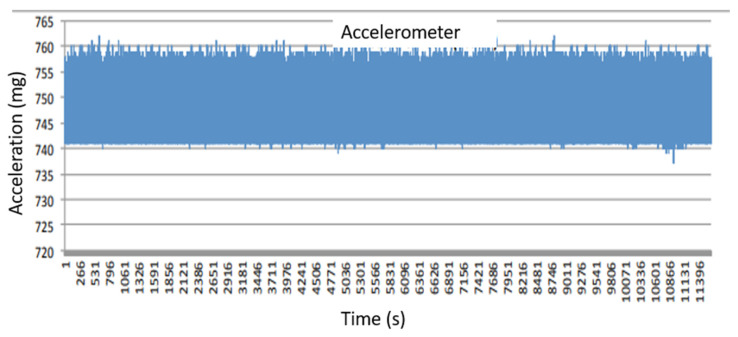
Evolution of the vibrations obtained during the measurement campaign.

The proposed solution arouses interest, considering its compactness and, at the same time, its capability to measure multiple physical quantities managed through the IoT approach. In particular, Figure 10 shows the temperature monitored inside the painting, whereas Figure 11 shows the behavior of relative humidity related to the artwork. The values obtained show that the temperature varies in the range of 27–31 °C and the relative humidity between 62% and 52%. Figure 12 evinces the vibrations measured inside the monastery and in particular the level applied into the painting. The values recorded to quantify the vibrations correspond to a variation of about 20 mg, which are related to minimal movements of the artifact and are therefore not a cause for alarm.

As already mentioned, the artwork under study is a canvas painting applied on a wooden support. The limits of both canvas paintings and wooden artefacts must therefore be taken into account when assessing microclimatic parameters. Considering Table 1, artwork could sustain variations between 17 °C and 26 °C for temperature and between 34–64% for relative humidity. The comparison with measured parameter trends for temperature (Figure 10) and relative humidity (Figure 11) seems to show that temperature exceeds the allowed value whereas the humidity remains in the accepted range. 

This test was of course only preliminary due to the reduced measurement time, and it was the first step to confirm the principle of the board for the aforementioned application. The work is in progress with an exhaustive study, in accordance with the regulations time.

## 4. Conclusions

In this work, a system for cultural heritage conservation is proposed based on a wireless IoT device for measurement of the microclimate parameters and Bluetooth^®^ low energy for data transmission. The device was characterized by using a climate chamber and an electrodynamic shaker to study its performances. Results demonstrated a good response function for all the three measured physical parameters. After the characterization performed in the laboratory, the system has been validated with a first test for the preventive conservation of an artifact, with the painting *The Deposition*, realized in the 19th century by Giuseppe Patricolo, placed inside a niche in the Santa Caterina Monastery in Palermo (Italy). The monitoring regarded microclimate parameters such as temperature, relative humidity and vibrations, which arouse interest for the preventive conservation and mechanical monitoring of the artifact under analysis. Results of this first test evince the suitability of the approach here pursued to measure the physical quantities of interest; in particular, measurements conducted in the monastery highlight the performance of the system and the obtained values show that the temperature varies in the range of 27–31 °C and the relative humidity between 62–52%. Monitoring is still active for an exhaustive study.

The proposed solution presents several novelties points considering the compactness, the low power budget, the robustness during operation and the ability to measure multiple measurand (temperature, relative humidity and vibrations) with the IoT method. It is worth mentioning that other already published works do not present all these prerogatives together. The work is in progress through the adoption of an evolution of the system here proposed with a scaling of the entire device and hybrid energy harvesters used to implement a fully autonomous measurement system for cultural heritage indoor and outdoor applications.

## Figures and Tables

**Table 1:** Temperature and relative humidity reference and deviation values for different categories of artifacts (from UNI10829:1999).

Materials and Objects of Organic Nature				
	Temperature (°C)		Relative Humidity %	
	Range	Deviation	Range	Deviation
Paper artifacts, papier-mâché, tissue paper, tapestries	18–22	1.5	40–55	6
Fabrics, velarium, carpets, tapestries, silk, costumes, clothing, religious vestments, natural fibers, sisal, jute *	19–24	1.5	30–50	6
Waxes, anatomical waxes	<18	NR	NR	NR
Herbaria and collections	21–23	1.5	45–55	2
Entomological collections	19–24	1.5	40–60	6
Animals, dried anatomical organs, mummies	21–23	1.5	20–35	-
Furs, feathers, stuffed animals and birds	4–10	1.5	30–50	5
Drawings, watercolors, pastels, and similar works on paper	19–24	1.5	45–60	2
Ethnographic collections, masks, leather, and leather clothing	19–24	1.5	45–60	6
Paintings on canvas, oil paintings on canvas, tempera, gouaches	19–24	1.5	40–55	6
Archival documents on paper and parchments, papyri, manuscripts, printed volumes, philatelic collections	13–18	-	50–60	5
Leather or parchment bindings	19–24	1.5	45–55	6
Lacquers, decorated or lacquered furniture	19–24	1.5	50–60	4
Polychrome wood sculptures, painted wood, paintings in wood, wooden icons, wooden musical instruments	19–24	1.5	50–60	4
Unpainted wooden sculptures, wicker objects, wooden panels or bark	19–24	1.5	45–60	4
**Materials and Objects of Inorganic Nature**				
Porcelain, ceramics, grès, terracotta, non-excavation tiles and excavated tiles if demineralized	NR	-	NR	10
Stones, rocks, minerals, stable (porous) meteorites	19–24	-	40–60	6
Stome mosaics, stones **, rocks, minerals, meteorites (non porous), fossils and stone collections	15–25	-	20–60	10
Metals, polished metals, metal alloys, silver, armour, weapons, bronze, coins, copper, tin, iron, steel, lead, pewter ***	NR	-	<50	-
Metals with active corrosion sites	NR	-	<40	-
Gold	NR	-	NR	-
Chalk	21–23	1.5	45–55	2
Unstable, iridescent, sensitive glass mosaics	20–24	1.5	40–45	-
**Mixed Objects**				
Wall paintings, frescoes, sinopites (detached)	10–24	-	55–65	-
Dry wall paintings (detached)	10–24	-	50–45	-
Ivories, horns, malacological, collection, eggs, nests, corals	19–24	1.5	40–60	6
Synthetic fibres	19–24	-	40–60	-
Film and photographs ****	0–15	-	30–45	-

* lower HR values are preferable for materials under tension. ** very specific relative humidity values (sample-dependent). *** in the case of objects made of different metal parts welded together, temperature fluctuations can be harmful. **** in the absence of specific manufacturer’s instructions.

**Table 2:** Characteristic of the sensors.

Device	Description	Main Performance	Size
STM32L4R9	Microcontrollori ARM-MCU Ultra-low-power FPU Arm Cortex-M4	- Operating voltage 1.71 V to 3.6 V - MCU 120 MHz 2048 kbytes of Flash USB OTG, DFSD	7 mm × 7 mm × 0.50 mm
STTS751	Low-voltage local digital temperature sensor	- Operating voltage 2.25 V to 3.6 V - Operating temperature −40 °C to +125 °C - Low supply current 3 μA (typical) standby - Accuracy ± 0.5 °C (typ) −40 °C to +125 °C	2 mm × 2 mm × 0.5 mm
HTS221	Capacitive Digital Humidity Sensor	- Operating voltage 1.7 to 3.6 V - 0% to 100% relative humidity range - Low power consumption: 2 μA @ 1 Hz - High RH sensitivity: 0.004% RH/LSB - Humidity accuracy: ±4.5% RH, 20% to +80% RH	2 mm × 2 mm × 0.9 mm
LIS2DW12	MEMS Digital Output Motion Sensor	- Ultra-low power consumption: 50 nA in power-down mode, below 1 µA in active low-power mode - Very low noise: down to 1.3 mg RMS in low-power mode - Supply voltage, 1.62 V to 3.6 V - ±2 g/±4 g/±8 g/±16 g full scale - 10000 g high-shock survivability	2.0 mm × 2.0 mm × 0.7 mm
